# Distributed Visual Crowdsensing Framework for Area Coverage in Resource Constrained Environments

**DOI:** 10.3390/s22155467

**Published:** 2022-07-22

**Authors:** Moad Mowafi, Fahed Awad, Fida’a Al-Quran

**Affiliations:** Department of Network Engineering and Security, Jordan University of Science and Technology, Irbid 22110, Jordan; fhawad@just.edu.jo (F.A.); fmalquran17@cit.just.edu.jo (F.A.-Q.)

**Keywords:** distributed visual crowdsensing framework, resource constrained environments

## Abstract

Visual crowdsensing applications using built-in cameras in smartphones have recently attracted researchers’ interest. Making the most out of the limited resources to acquire the most helpful images from the public is a challenge in disaster recovery applications. Proposed solutions should adequately address several constraints, including limited bandwidth, limited energy resources, and interrupted communication links with the command center or server. Furthermore, data redundancy is considered one of the main challenges in visual crowdsensing. In distributed visual crowdsensing systems, photo sharing duplicates and expands the amount of data stored on each sensor node. As a result, if any node can communicate with the server, then more photos of the target region would be available to the server. Methods for recognizing and removing redundant data provide a range of benefits, including decreased transmission costs and energy consumption overall. To handle the interrupted communication with the server and the restricted resources of the sensor nodes, this paper proposes a distributed visual crowdsensing system for full-view area coverage. The target area is divided into virtual sub-regions, each of which is represented by a set of boundary points of interest. Then, based on the criteria for full-view area coverage, a specific data structure theme is developed to represent each photo with a set of features. The geometric context parameters of each photo are utilized to extract the features of each photo based on the full-view area coverage criteria. Finally, data redundancy removal algorithms are implemented based on the proposed clustering scheme to eliminate duplicate photos. As a result, each sensor node may filter redundant photographs in dispersed contexts without requiring high computational complexity, resources, or global awareness of all photos from all sensor nodes inside the target area. Compared to the most recent state-of-the-art, the improvement ratio of the added values of the photos provided by the proposed method is more than 38%. In terms of traffic transfer, the proposed method requires fewer data to be transferred between sensor nodes and between sensor nodes and the command center. The overall reduction in traffic exceeds 20% and the overall savings in energy consumption is more than 25%. It was evident that in the proposed system, sending photos between sensor nodes, as well as between sensor nodes and the command center, consumes less energy than existing approaches due to the considerable amount of photo exchange required. Thus, the proposed technique effectively transfers only the most valuable photos needed.

## 1. Introduction

People nowadays use smartphones extensively in their daily lives. More and more crowdsensing solutions are being researched as a result of advanced sensing capabilities integrated within mobile devices. Visual crowdsensing (VCS) applications, which employ the built-in cameras of smartphones to provide more detailed information (i.e., images or videos) about the sensing region, have recently attracted the attention of respective research groups [1]. Visual data has been incorporated into a wide range of new applications, including visual tracking and the Internet of multimedia things. Visual information, based on the extensive knowledge that can be gathered from it, can undoubtedly influence the performance of many applications. As a result, VCS has broadened the range of mobile crowdsensing applications. Flexsensing [2], LuckyPhoto [3], SmartPhoto [4], Picpick [5], FlierMeet [6], and CrowdTracking [7] are examples of previous projects that demonstrate that VCS is useful and, in many cases, superior to traditional visual sensing solutions that rely on the deployment of stationary cameras for monitoring.

The VCS paradigm differs from traditional mobile crowdsensing in its nature. When opposed to scalar data, visual data typically requires more data processing and bandwidth to convey. Furthermore, data redundancy is regarded as one of the most significant issues in VCS. For example, in disaster recovery applications, making the most out of limited resources to collect the most useful photos from the public is a challenge. Methods for detecting and eliminating redundant data provide a number of advantages, including lower transmission costs and overall energy consumption. 

There are two types of redundancy in data: content redundancy and context redundancy. Visual similarity is quantified in content redundancy using visual features and color histogram [8], whereas, in context redundancy, it is quantified using location or shooting angle information. Moreover, the geographical and geometrical details are utilized to check the similarity of the photographs in context redundancy. For example, different buildings may have similar visual features, but there is no context resemblance since their locations are different. In [9,10], content photodetection algorithms were used to confirm the similarity of photo contents. On the other hand, mobile devices have limited computing capacity and energy to run such algorithms in disaster recovery circumstances. As a result, context-based solutions are more suitable for disaster recovery scenarios.

Nonetheless, depending on the requirements of the VCS applications, redundant context data detection may vary according to the type of visual information that needs to be covered. One-way coverage of the point of interest (PoI) is required in classical visual sensor networks. In some cases, multiple aspects or *k*-views of the same PoI should be observed from various directions. In other words, redundancy detection is dependent on the type of coverage required in the VCS application. For instance, if the goal is to cover the building regardless of which direction it is covered and there are two photographs of the same building (i.e., content similarity), then these two images are redundant. They may not be redundant, however, if the task needed is to provide a full view of the building. Previous works [11,12] presented a framework for a full view of PoIs, in which each PoI within the target area must be covered from all directions. On the other hand, the authors of [13] developed a framework for full-view sub-regions, in which each sub-region should be covered from all directions. It is worth noting that most existing VCS systems are proposed for full-view point coverage [13]. On the other hand, the existing systems for full-view area coverage use a centralized framework, assuming all sensor nodes in the network communicate with a server that uses a photo redundancy elimination method based on global knowledge of the geometric locations of all nodes’ photos. In VCS, there is currently no structure for removing duplicates between photographs based on full-view area coverage. 

On the other hand, VCS solutions are crucial in disaster assistance or emergency circumstances since they provide real-time and detailed information about the target area. VCS systems should appropriately address various limitations in such critical situations, including restricted bandwidth and disrupted communication with the command center or server. 

In this paper, we present a distributed VCS system for full-view area coverage that addresses interrupted communication with the command center. The nodes within the target area make use of free transmissions such as Wi-Fi and Bluetooth to share photos. The nodes not only store their own acquired images but they may also store shared images received from other sensor nodes, which duplicates and expands the amount of data stored on each sensor node. As a result, if any node can communicate with the server, then more photos of the target region will be available to the command center. This condition allows the command center to learn more about the target area and, as a result, take appropriate actions to deal with difficult situations. For such scenarios, many routing schemes were proposed, such as routing protocols in delay-tolerant networks [11,12] and mobile opportunistic networks [14].

The contributions of this work can be summarized as follows:

1. Proposing a distributed VCS framework for full-view area coverage in resource-constrained environments. In the proposed approach, the target area is divided into virtual sub-regions, each of which is represented by a set of boundary points of interest (BoI). An appropriate pattern of BoIs that can be used to investigate the full-view area coverage is found. The area is full-view covered if each BoI has full-view coverage in all *k*-view directions around the full scope. To the best of our knowledge, this is the first distributed framework in VCS to investigate full-view area coverage by utilizing a set of BoIs rather than the target region’s continuous domain or using dense grid methods. Because there are infinite points inside the target area, mobile devices have limited processing ability and energy to investigate the area coverage utilizing the continuous domain of the target region. The BoI scheme is proposed to investigate the full-view area coverage for applications with limited resources for this aim.
2. Developing a clustering scheme to represent the geometric coverage conditions of each photo in VCS, based on the full-view area coverage conditions used in traditional camera sensor networks (CSN). Although there are data redundancy removal algorithms for full-view area coverage in CSN [15,16], these approaches are ineffective for VCS since users take different images from different positions each time. For instance, the authors in [15] proposed an approach that considers fixed locations of sensors and thus performs data redundancy elimination based on predefined locations.
3. Developing adaptive photo redundancy elimination procedures based on the coverage of each photo in the target area. This allows the distributed VCS system to select the minimum number of photos with maximum area coverage. As a result, each sensor node can filter redundant photos without requiring high computational capabilities or a global understanding of all photos from all sensor nodes inside the target area. The proposed procedures prevent sending repeated photos across sensor nodes, which saves bandwidth and energy.

## 2. Related Works

There are many applications of VCS, such as environmental monitoring, scientific data collection, public information gathering, and target area sensing [1,6,17]. In VCS, visual data typically requires more data processing and bandwidth to upload photos. In [13], a centralized VCS solution for full-view area coverage is proposed to address this issue. The metadata for all current photographs is uploaded first by the sensor nodes. The server next analyzes the photographs’ metadata to determine the overall photo utility (i.e., how much and to what degree each photo covers the target region) and which set of photos should be uploaded to the server. They first used a centralized procedure to divide the coverage area of all images into sub-regions, with each sub-region being covered by the same collection of photos as in prior works [15]. The server then selects photographs with higher photo utility value and only requests valuable photos from the nodes using the utility area function. As a result, nodes upload the images with the highest added value and reduce the uploading of redundant photos. Similar to [13], a multi-coverage picture collection framework was proposed in [18] as a server-side scheme. They devised a pyramid-tree-based approach for dynamically clustering photographs and adapting to crowd-captured photo changes. To discover redundant photographs, the server does dynamic clustering of photos. Using the Ptree model, each photo is represented by a clustering object using a series of semantic and visual criteria to remove redundant photos. The work in [19] is similar to [13], where the metadata of the photos is first sent to the server and then the server analyzes the photos and selects the most valuable photos. All the proposed schemes in [13,18,19] adopted a centralized approach and considered the availability of constant contact between the nodes and the server side, which is not the ideal case in emergency scenarios such as a disaster.

Several distributed systems were proposed to address the interrupted contact with the server side. In [12], a PicTree model is generated by each node to cluster its photo storage. When two nodes get to communicate with each other, each one should share its PicTree with the other. The tree fusion mechanism was proposed to allow the PicTrees of the two nodes to merge. They can cluster the photographs and remove the redundant photos between the two sensor nodes after fusing the two PicTrees of the two sensor nodes. The redundant photographs are those that match in all layers and have the same features. As a result, each node knows which photo needs to be sent to the other node and which picture needs to be received. In [20], the authors proposed a distributed framework for processing images captured by crowds within the target area following a natural disaster. The goal of this work is to reduce the overhead of processing photos on the server side. They, however, classified the photos based on their content features using image processing, which required more processing power. The work in [11] is an example of distributed photo coverage in VCS using a fixed list of PoIs. For a dynamic list of PoIs, the authors in [21,22] proposed distributed photo crowdsourcing in delay-tolerant networks. They considered the changing and dynamic nature of events, and as a result, they offered a photo framework selection that adapts to changing list of PoIs. They included a mechanism for adding new PoIs, updating existing PoIs, and removing existing PoIs. However, they considered the whole view of points but not the full-view coverage of the area. The main challenge is to determine whether the target area is covered from all directions. 

The full-view coverage area was lowered to full-view coverage of points in CSN. In [15], the authors looked at the geometric metadata of the camera nodes as well as the geometric full-view coverage area conditions inside the target area to divide it into sub-regions, each with an identical combination of sensors and coverage conditions. Then, they looked at the full-view coverage conditions of each segment in the sub-boundary region that came out of the partitioning operation. They derived a set of points from each segment in the sub-boundary region that ensure full-view coverage. However, the detection approach for a full-view area coverage of each border segment requires global knowledge of the present set of CSN. Moreover, any change to the current camera sensor deployment would drastically alter the area’s coverage circumstances, and therefore the set of points that guarantee full-view area coverage would be altered. Existing works in CSN that convert the difficulty of researching the target area into studying the points are still incompatible with distributed VCS solutions. In [16], the authors were able to choose the smallest number of sensor nodes with the least amount of uncovered space between them. It was assumed that there is a central point that knows the locations of all nodes in the network and chooses the best location for the sensor nodes to provide the best coverage area. Nevertheless, in a distributed context, there is no central point to locate the sensor nodes positions that provide the best cover. 

## 3. Methodology

The proposed framework for full-view area coverage is based on transforming the problem of the area covered in the continuous domain into an area coverage of boundary points. This required the development of the following subsystems:

1. A boundary points of interest model to generate the appropriate pattern for full-view area coverage.
2. A classification method to represent the *k*-view geometric area coverage conditions. This subsystem collects and classifies photos with the same *k*-view geometric area coverage features with the application of the BoIs model in a distributed and adaptive manner.
3. A set of procedures to eliminate redundancy among photos with identical geometric conditions for VCS. This includes the management of the sensor node storage to prevent storing redundant photos and how each sensor node stores photos with maximum added values so that photos with higher coverage overlap are skipped.

### 3.1. Proposed Model for Boundary Points of Interest

To study the full-view area coverage, instead of using the continuous domain of the target area [13] or the dense grid model [23], the target area is divided into virtual sub-regions represented with sets of boundary points called BoIs. When converting this problem into VCS, each virtual sub-region is considered full-view-covered only when the BoIs of each sub-region are full-view-covered by a specific set of photos.

**Figure 1:**
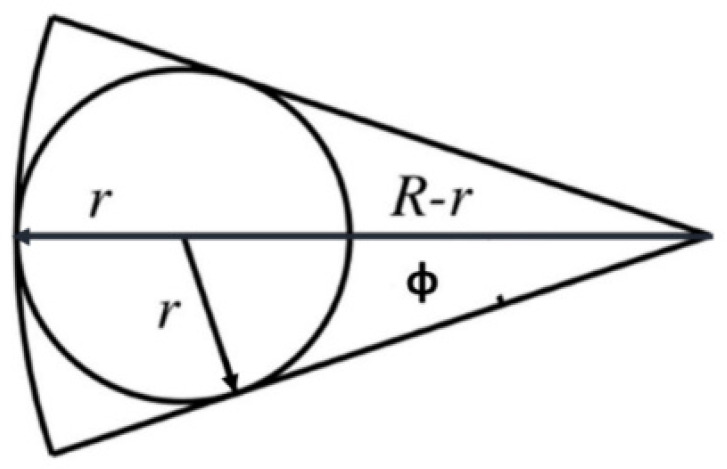
Maximum coverage disk of a camera sensor.

**Figure 2:**
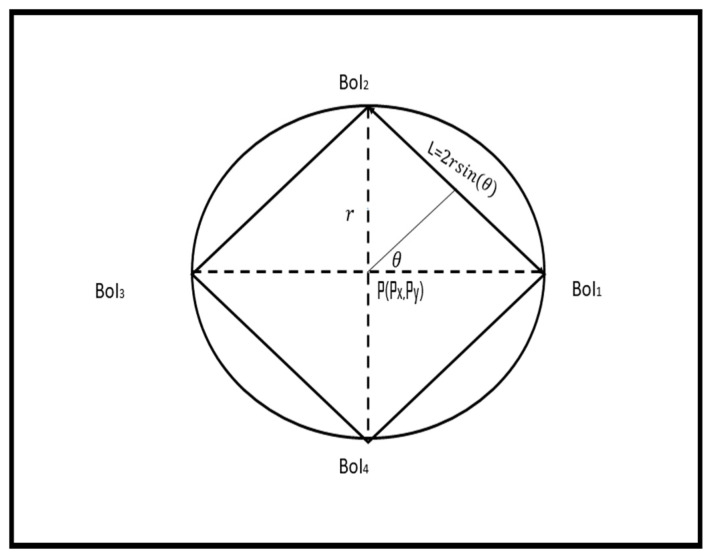
Maximum sub-region inside the maximum coverage disk.

To study the full-view area coverage, the appropriate distance between the BoIs needs to be extracted. The coverage range is influenced by the shooting angle, the field of view (FoV), and the location of the camera sensor. Figure 1 shows the coverage range of a camera sensor. The maximum coverage area is represented by a disk with radius *r*, which can be expressed as:

(1) $$r=R\times \frac{\sin\left(\Phi \right)}{1+\sin\left(\Phi \right)}$$

where Φ is half the FoV and *R* is the sensing range of the camera. The maximum coverage disk is examined by a circular set of camera sensors, considering the circumstances described in [16]. Using the maximum coverage disk, we can determine the maximum unit area (or sub-region) within the disk that is full-view covered by a specific set of photos. This sub-region is represented by a set of BoIs located at the circumference of the maximum coverage disk. Figure 2 shows the maximum square area (sub-region) within the maximum coverage disk with four BoIs. Given the maximum coverage disk radius, *r*, the length of the square sub-region, *l*, can be obtained as follows:

(2) $$\sin\left(\theta \right)=\frac{l}{2r}$$

(3) l=2r×sin(θ)

Algorithm 1 illustrates the procedure for creating the pattern of BoIs for full-view area coverage.
**Algorithm 1:** BoIs generation1: Determine the length of the target area 2: Determine the width of the target area 3: Determine the radius of maximum coverage disk (*r*)4: Sub-region area = (2*r* × 2*r*)/2 5: Number of sub-regions = Target area/Sub-region area6: *S*: Total Number of sub-regions7: *B*: Number of BoIs for each sub-region8: *P_x_*(*s*): location-*x* center of sub-region (*s*)9: *P_y_*(*s*): location-*y* center of sub-region (*s*) 10: **for**
*s* = 1:*S*
**do**11:  **for**
*b* = 1:*B*
**do**12:      BoI*x*(*s*, *b*) = *Px*(*s*) + *r* × cos((*b* − 1) × (360/*B*))13:      BoI*y*(*s*, *b*) = *Py*(*s*) + *r* × sin((*b* − 1) × (360/*B*))14:  **end for**15: **end for**

To achieve full-view area coverage, all the points within each sub-region should share the following full-view geometric coverage conditions [13,16]:

1. The points within each sub-region are covered by the same set of photos.
2. The photos covering the same sub-region can be ordered in a clockwise or anti-clockwise direction depending on their positions with respect to the sub-region.
3. The rotation angle between any two neighboring sensors within the same set and any point within the sub-region is either within the safe or unsafe region.

An area is considered a safe region if the rotation angle between any two neighboring sensors and any point inside the area is less than 2*θ*. If the rotation angle between any two neighboring sensors and any point inside the area is more than 2*θ*, the area is considered an unsafe region.

**Figure 3:**
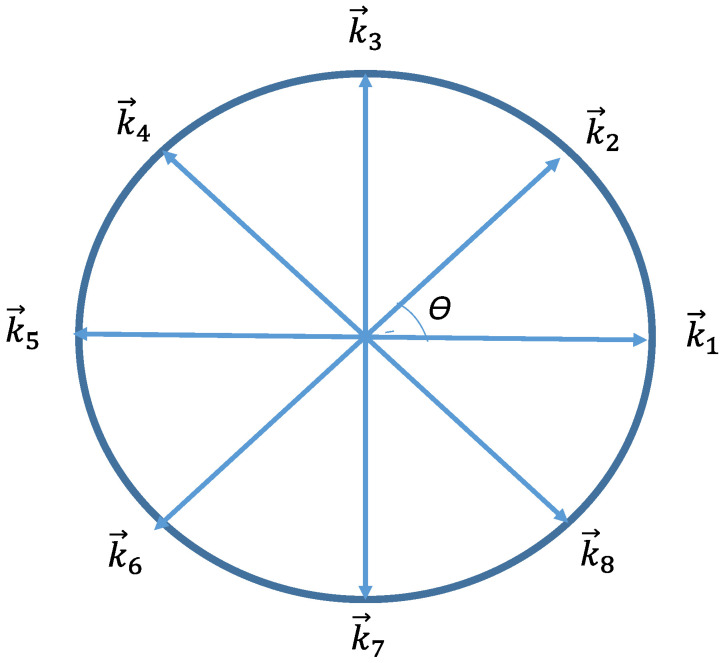
The division of the full scope around the BoI.

In the proposed model, the entire scope around the BoI is split into *k* sectors, where *k* = 2π/*θ*, as shown in Figure 3 with *k* = 8 and *θ* = 45 degrees. In Figure 3, vector ${\vec{k}}_{1}\;$ denotes angle 0 while $\;{\vec{k}}_{2}$ denotes angle *θ*, until ${\vec{k}}_{\left(2\pi ∕\theta \right)}$ is attained. Accordingly, sector 1 has the angle range [0, 45], sector 2 has the angle range [45, 90], until sector 8, which has the angle range [315, 360]. A BoI is considered covered from the direction $\vec{k}$ if the rotation angle between $\vec{k}$ and the vector from the camera location towards the BoI ($\vec{SB}$) is smaller than or equal to *θ*. Hence, sector 1, for instance, is considered covered with the following criteria:

1. The rotation angle between ${\vec{k}}_{1}$ and $\vec{SB}$ is smaller than or equal to *θ*.
2. The rotation angle between ${\vec{k}}_{2}$ and $\vec{SB}$ is smaller than or equal to *θ*.

### 3.2. Proposed Photo Clustering and Selection Scheme

To determine the set of photos that cover the same sub-region under the same geometric coverage conditions, a layer-based tree, called K-CoverPicTree, for clustering the photos is proposed. This is to assure the appropriateness of the geometric coverage conditions of each photo during the examination of the full-view area coverage in a distributed manner within a visual crowd system. 

The K-CoverPicTree consists of the following layers:

1. The first layer comprises the ID of the sub-regions. The partitioning process is simplified by using photos belonging to the same sub-region ID.
2. The second layer comprises all BoIs of each sub-region.
3. The third layer comprises the ID of each covered sector for each covered BoI within each sub-region. This layer determines if all sectors around each BoI within the same sub-region are fully covered by the same set of photos.
4. The fourth layer comprises the ID of each photo of all covered sub-regions, BoIs, and sectors. Each photo has a unique ID in terms of location, FoV, shooting angle, and sensing range.

The sectors around each BoI have a uniform order as the sector ID starts from angle 0. This angle denotes the vector pointing to the right (east on the map), and the sector ID is increased by 1 in the counterclockwise direction for each *θ* until 360 degrees is reached (see Figure 3). The photos can therefore be ordered in terms of their sub-region following the order of the covered sectors. In each sub-region, the order of the BoIs has a direction similar to that of the proposed BoIs model. As an example, suppose that a photo with ID = *e* (photo *e*) covers sector 3 (from angle 90 to 135) for all BoIs with sub-region ID = 1. Photo *d* covers sector 2 (from angle 45 to 90) for all BoIs with sub-region ID = 1. Photo *c* covers sector 1 (from angle 0 to 45) for all BoIs with sub-region ID = 1. Photo *f* covers sector 4 (from angle 135 to 180) for all BoIs with sub-region ID = 1. For such a situation, the photo set can be ordered from sector 1 to sector 4 as photos *c*, *d*, *e*, and *f*, with sub-region ID = 1. The ordered photos would affirm whether the rotation angle between any two neighboring photos and any BoI is smaller than 2*θ*. In addition, a gap between the order of covered sectors of a BoI means that the BoI is within the unsafe region according to condition 3 of the full-view geometric coverage [13]. Otherwise, the BoI would be within the safe region. Here, the rotation angle between any two neighboring photos is smaller than 2*θ*. Condition 3 requires that all the BoIs within the same region be in the safe region so that the BoIs fulfill the full-view coverage of the sub-region. The photo order, therefore, needs to be found first. Considering the previous example of the photo set *c*, *d*, *e*, and *f*. These photos cover sectors 1 to 4 of all BoIs with no gap, and accordingly, they cover all scopes, from 0 to 180 degrees of sub-region 1.

The formation of the initial K-CoverPicTree is denoted by Algorithm 2. First, each sensor inside the target area generates an empty K-CoverPicTree. Initially, there are three layers where the first layer comprises all sub-region IDs of the target area, as can be viewed in line 4. The second layer comprises the IDs of every BoI for each sub-region inside the target area, as can be viewed in line 6. The third layer comprises the sector IDs for all BoIs within every sub-region inside the target area, as can be viewed in line 8. Captured photos are all appended to the tree following the sequence values of the covered sub-region ID, the BoI ID, and the sector ID (*s*, *b*, *k*). 

The sequence of coverage features (sub-region ID, BoI ID, and sector ID) or (*s*, *b*, *k*) are extracted for each photo using the proposed BoI model. For example, suppose that photo *a* covers sector 1 (from angle 0 to 45) of BoI 1 and covers sector 1 of BoI 2 of sub-region 1, and that photo *b* covers sector 3 (from angle 90 to 135) of BoI 1, and sector 3 of BoI 2 of sub-region 1. Accordingly, photo *a* will have the sequences: (*s* = 1, *b* = 1, *k* = 1) and (*s* = 1, *b* = 2, *k* = 1) and photo *b* will have the sequences: (*s* = 1, *b* = 1, *k* = 3) and (*s* = 1, *b* = 2, *k* = 3).
**Algorithm 2** Initial K-CoverPicTree generation1: Input *S*: number of virtual sub-regions2: Input *B*: number of BoIs for each sub-region3: Input *K*: number of sectors for each BoI4:  **for**
*s* = 1:*S*
**do**5:  Node(*s*) = *s*;6:  **for**
*b* = 1:*B*
**do**7:      Node(*s*, *b*) = *b*;8:      **for**
*k* = 1:*K*
**do**9:       Node(*s*, *b*, *k*) = *k*;10:       **end for**11:   **end for**
12: **end for**

### 3.3. Data Redundancy Elimination

In the proposed system, each sensor node manages local knowledge of the photos in its local K-CoverPicTree. In the generation of a new photo, there are no redundant photos being stored with the same coverage conditions in accordance with the local set of existing photos within each sensor. The following sections describe how each sensor node manages its stored set of photos and how the sensor nodes share their local K-CoverPicTree between each other and prevent transmission of redundant photos. 

#### 3.3.1. Local Data Management

Each sensor node manages the local information of the existing set of photos using the proposed clustering scheme. Each node creates two local trees as follows:

1. Local K-CoverPicTree: The initial local K-CoverPicTree is produced with the application of Algorithm 2. This tree is used in the classification and clustering of the existing set of photos within the local storage of each sensor.
2. Local Boolean K-CoverPicTree: Prior to the addition of photos to the local K-CoverPicTree, the added value of each photo is examined by each sensor with the application of the local Boolean K-CoverPicTree. Within this tree, the index of each node comprises the sub-region ID, BoI ID, and sector ID.

Each node in the local Boolean K-CoverPicTree carries the value of 0 or 1, where 0 means no photo covering a specific sequence of (*s*, *b*, *k*) while 1 denotes the existence of a photo covering a specific sequence of (*s*, *b*, *k*). All nodes are initialized with the value of 0. If the local K-CoverPicTree has a child photo for Node(*s*, *b*, *k*), then the value of the corresponding Boolean K-CoverPicTree node, Bool_Node(*s*, *b*, *k*), will change from 0 to 1. Algorithm 3 shows how the added value for each photo is computed.
**Algorithm 3** Counting the added value of a photo1: Extract the metadata of the photo2: Extract the sequence of coverage features values (*s*, *b*, *k*) of the photo3: Added value = 0;4: **for** each coverage sequence (*s*, *b*, *k*) in the local Boolean K-CoverPicTree **do**5:      **if** Bool_Node(*s*, *b*, *k*) = 0 **then**
6:       Added value = Added value + 17:      **end if**8: **end for**

The application of the Boolean K-CoverPicTree begins with the examination of the added value of each photo prior to adding the photo to the local K-CoverPicTree. A photo is added to the local K-CoverPicTree when its added value is greater than zero. However, if the newly generated photo has an added value of zero, then each sensor will search within its local K-CoverPicTree and check whether there exists a photo with the same features of coverage values (sub-region ID, BoI ID, and sector ID) or whether the coverage features of the photo entails a sub-set of the coverage features of the existing photos. In this case, the sensor will exclude the photo and consider it redundant. On the other hand, if there is no other photo with the same features of coverage values or sub-set of the features of coverage values, the sensor will add the photo to the local K-CoverPicTree with the respective node index.

As an example, suppose that photo *a* covers the sequences (*s* = 1, *b* = 1, *k* = 1), (*s* = 1, *b* = 2, *k* = 1) and (*s* = 1, *b* = 3, *k* = 1). In addition, photo *b*, which resides within the local K-CoverPicTree, covers the sequences (*s* = 1, *b* = 1, *k* = 1), (*s* = 1, *b* = 2, *k* = 1), (*s* = 1, *b* = 3, *k* = 1) and (*s* = 1, *b* = 4, *k* = 1). It can be stated that photo *a* is redundant in relation to photo *b*. This owes to the fact that the sequences (1, 1, 1), (1, 2, 1) and (1, 3, 1) are subset of (1, 1, 1), (1, 2, 1), (1, 3, 1), and (1, 4, 1).

**Figure 4:**
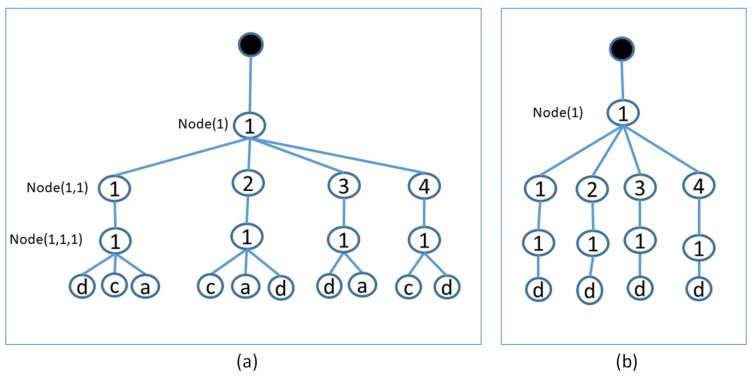
Example of (**a**) adding photos and (**b**) removing redundant photos from the local K-CoverPicTree.

To demonstrate the advantages of the local trees, suppose that a new photo is created on the sensor each time. Figure 4a shows the local K-CoverPicTree after adding four photos (*a*, *b*, *c*, and *d*). Here, Algorithm 2 is applied to form the initial local K-CoverPicTree and the initial local Boolean K-CoverPicTree is generated. The details are as follows:

1. A newly produced photo with ID = *a* (photo *a*) has the following coverage values sequence: (*s* = 1, *b* = 1, *k* = 1), (*s* = 1, *b* = 2, *k* = 1), and (*s* = 1, *b* = 3, *k* = 1). In this regard, utilizing Algorithm 3, the added value of photo *a* is 4. As the added value of the photo is higher than 0, the photo is appended to the local K-CoverPicTree. This is followed by the update of the value of the local Boolean K-CoverPicTree nodes (1, 1, 1), (1, 2, 1), and (1, 3, 1) to 1.
2. A newly produced photo with ID = *b* (photo *b*) has the following coverage values sequence: (*s* = 1, *b* = 1, *k* = 1), (*s* =1, *b* = 2, *k* = 1), and (*s* =1, *b* = 3, *k* = 1). Utilizing Algorithm 3, the added value becomes 0. In the next step, the sensor searches in its local K-CoverPicTree to ascertain whether there exists a photo sharing a similar sequence of coverage features. As photo *b* covers a similar sequence of coverage features (*s*, *b*, *k*) as photo *a*, it is regarded as redundant.
3. A newly produced photo with ID = *c* (photo *c*) has the following coverage sequence: (*s* = 1, *b* = 4, *k* = 1), (*s* = 1, *b* = 1, *k* = 1), and (*s* = 1, *b* = 2, *k* = 1). Utilizing Algorithm 3, the added value is 1. Photo *c* is appended to the local K-CoverPicTree, and following the new addition, the local Boolean K-CoverPicTree node (1, 4, 1) is revised as 1.
4. A newly produced photo with ID = *d* (photo *d*) has the following sequence of coverage features: (*s* = 1, *b* = 1, *k* = 1), (*s* = 1, *b* = 2, *k* = 1), (*s* = 1, *b* = 3, *k* = 1) and (*s* =1, *b* = 4, *k* = 1). For photo *d*, the added value is 0. However, a photo with similar sequence of coverage features does not exist, and thus, photo *d* is appended to the local K-CoverPicTree.

Adding photo *d* makes the photo *a* redundant because its coverage sequence values are a subset of the coverage sequence values of photo *d*. Furthermore, this makes photo *c* redundant because its coverage sequence values are a subset of the coverage sequence values of photo *d.* Figure 4b shows the final state of the local K-CoverPicTree after removing photo *a* and photo *c*. Hence, each sensor can compute the redundant photos according to the full-view coverage area conditions. 

The steps to remove redundant photos are shown in Algorithm 4. First, in line 3, photos are filtered to find those that cover sub-region *s*, Photos(*s*). Then, all photos that cover each BoI in sub-region *s* from the same sector view, Photos(*s*, *b*, *k*), are found, as stated in line 6. Then, in line 8, for each sector of value *k*, photos covering the entire sub-region (i.e., all BoIs within the sub-region) Photo(*s*, *k*) are found. Redundant photos are then determined by subtracting Photos(*s*, *k*) from Photos(*s*, *b*, *k*), as in line 11. This way, the sensor node keeps a log of photos with higher added value and deletes the photos that are redundant. 

In Figure 4, photo *d* covers (*s* = 1, *b* = {1, 2, 3, 4}, *k* = 1), while photo *c* covers (*s* = 1, *b* = {1, 2, 4}, *k* = 1). It appears that photo *c* covers sub-region 1. However, the photo does not correspond to line 8 because it does not cover all BoIs of sub-region 1. Using lines 10 to 12, photo *a* and photo *c* are removed.
**Algorithm 4** Removing redundant photos1:    Input: Local K-CoverPicTree2:    **for**
*s* = 1:*S*
**do**3:   Find all photos that cover sub-region *s* (Photos(*s*))4:   **for** *k* = 1:*K*
**do**5:     **for** *b* = 1:*B*
**do**6:       Find photos that cover (*s*, *b*) from the same sector view (Photos(*s, b, k*))7:     **end for**8:     Find photos that cover all BoIs of sub-region *s* from sector *k* (Photos(*s*, 1:*B*, *k*) = Photos(*s*, *k*))9:   **end for**10:    **if** (size of Photos(*s*, *k*) > 0) **do**11:      Redundant Photos = Photos(*s*, *b*, *k*) − Photos(*s*, *k*)12:      Remove each redundant photo from the corresponding parent Node(*s*, *b*, *k*) in Local K-CoverPicTree13:    **end if**14: **end for**

Furthermore, each sensor node can compute the utility of its photos on the target area based on the final state of the local K-CoverPicTree. Accordingly, the steps to be taken in the computation of the utility value of the photos are shown in Algorithm 5. First, the initial value of the total utility is set to zero. Then, Algorithm 4 is called for finding the photos covering sub-region *s* from the same sector view. For each sub-region *s* inside the target area, if the size of Photos(*s*, *k*) > 0, the total utility is updated as shown in line 7, as the utility of Photos(*s*, *k*) is added to the total utility value. The utility of Photos(*s*, *k*) is the area of sub-region *s* multiplied by *θ*, where *θ* is the effective angle. Steps 6 and 7 are repeated for each sub-region *s* in the target area. As an example, sub-region 1 is regarded as full-view covered, providing that the utility of sub-region 1 reaches 2π × area of sub-region 1.
**Algorithm 5** Calculating the utility of the photos 1:    Input: Local K-CoverPicTree2:    Total utility = 03:    Call Algorithm 44:    **for** s = 1:*S*
**do**5:    **for** k = 1:*K*
**do**6:     **if** ((size of Photos(*s, k*) > 0)) **then**7:       Total utility = Total utility + Area of sub-region × *θ*8:     **end if**9:    **end for**10:   **end for**

The following is the general sequence of the proposed framework within each sensor:

1. In accordance with Algorithm 3, using the Boolean K-CoverPicTree, the added value of each photo is computed.
2. The photo with the highest added value is appended to the local K-CoverPicTree. Then, the Boolean K-CoverPicTree is revised based on the new inclusion as updates to the values of *s*, *b*, and *k* of the photos from values 0 to 1 in the Boolean K-CoverPicTree.
3. In the situation where the highest added value is zero, the next step is to look for photos with a similar sequence of coverage values. Alternatively, the subset of the coverage values of other available photos within the local K-CoverPicTree can be searched. In this regard, if photos with a similar sequence of coverage values or the subset of the coverage values cannot be found, the photo should be appended to the local K-CoverPicTree.
4. Then, utilizing Algorithm 3, the added value of the remaining photos in the device storage is calculated again. The previous steps are repeated until the set of photos is empty.
5. Utilizing Algorithm 4, redundant photos are removed.

#### 3.3.2. Communication between Two Nodes

**Figure 5:**
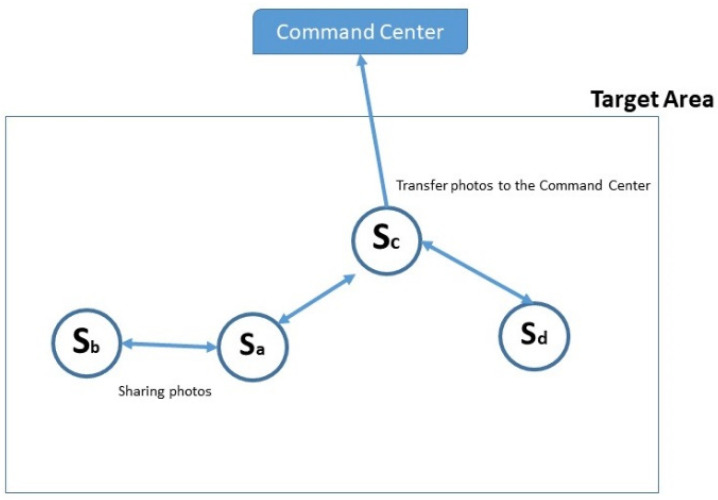
An example of a communication pattern used in the proposed system.

Within disruption tolerant networks (DTN), there is no constant contact between all sensor nodes and the command center or server [11]. The sensors leverage free links, including Wi-Fi and Bluetooth, to transfer photos between each other. When a sensor node detects a peer in its communication range, contact is happening between the two sensor nodes. The target of sharing photos between nodes is to increase the amounts of photos on each node. Hence, when communication occurs between any node and the server, more photos (i.e., more information) become available. An example of the communication pattern used in the proposed system is shown in Figure 5. Sensor nodes *a*, *b*, *c*, and *d* are located within the target area. Only sensor node *c* has access to the command center. However, sensor nodes *a* and *b* are in the communication range of each other, allowing photos to be shared between sensor node *a* and sensor node *b*. After receiving photos from sensor node *b*, sensor node *a* can send photos from its storage to the command center through sensor node *c*. Sensor node *a* can now store photos from sensor node *b* in addition to its own. In this manner, the command center is able to obtain photos from any connected sensor node inside the target area.

To simulate the interrupted contact with the server, it was assumed that 15 randomly selected sensor nodes out of the total nodes could contact and transmit photos to the command center. When sensor node *a* receives photos from other sensor nodes, it records the information of the source sensor node and the last time of contact with the source. Sensor node *a* then estimates the probability that it has encountered another sensor node within time *t*: *P*(*T_a_* ≤ *t*) = 1 − e^−λ^_a_*^t^*, where *T_a_* is the inter-contact time between sensor node *a* and other sensor nodes and λ_a_ represents the contact frequency between sensor node *a* and other sensor nodes [11]. In the local K-CoverPicTree, photos are removed if *P*(*T_a_* ≤ *t*) is higher than a specified threshold [11]. Hence, each node should manage the cache memory of its photos. First, it calculates the time, *t*, elapsed from the last contact with other sensors. This is followed by the calculation of the *P*(*T_sensor_* ≤ *t*). If *P*(*T_sensor_* ≤ *t*) is higher than a threshold of 0.8, a sensor removes all photo IDs from its local K-CoverPicTree. Algorithm 6 is applied to update the Boolean KCoverPicTree. This step is important to update the cache memory of each sensor node and prevent storing old photos.

When two sensor nodes *a* and *b* make contact, the sensor node’s delivery probability to the command center is computed, as defined in [24]. The possibility of delivering photographs to the command center is increased if the communication between sensor node *a* and the command center is frequent. We assume sensor node *a* has the highest probability of delivering the photos to the command center, resulting in the following general sequence:

1. Sensor nodes *a* and *b* share the initial local K-CoverPicTree with each other without photos transmission.
2. Each sensor node then searches for Photo IDs that share similar sub-regions IDs between its local K-CoverPicTree and the local K-CoverPicTree of the other sensor nodes. In this regard, the sensor node processes only the photos with common sub-region IDs between the two sensors. In other words, within the target area, only certain photos from certain sub-region IDs are processed, resulting in the reduction of the overhead of transmission of photos associated with all sub-regions within the network. Each sensor node finds the common sub-regions (S-common) between its local K-CoverPicTree and local K-CoverPicTree of other sensor nodes.
3. Sensor node *a* applies Algorithm 6 to merge its local K-CoverPicTree with the local K-CoverPicTree of sensor node *b* according to the shared sub-regions (S-common). As shown in lines 5–7, all branches of common sensor nodes in the local K-CoverPicTree of sensor node *b* are merged by sensor node *a* to its local K-CoverPicTree, resulting in a new tree called New Combined Tree(*a*). As shown in line 9, sensor node *a* identifies the newly added photos through the identification of the difference between the New Combined Tree(*a*) and its local K-CoverPicTree. Sensor node *a* then requests the required photos from sensor node *b* and updates its local K-CoverPicTree as shown in line 10.
4. Sensor node *b* applies similar steps to Algorithm 6 to merge its local K-CoverPicTree with the local K-CoverPicTree of sensor node *a* according to the shared sub-regions (S-common). Sensor node *b* merges all branches of common sensor nodes of the local K-CoverPicTree of sensor node *a* to its local K-CoverPicTree, resulting in the New Combined Tree(*b*). The newly appended photos are computed by sensor node *b* through the difference identification between the New Combined Tree(*b*) and its local K-CoverPicTree. Sensor node *b* then requests the required photos from sensor node *a* and updates its local K-CoverPicTree.
5. Finally, sensor nodes *a* and *b* update their local Boolean K-CoverPicTree, after adding the requested photos from each other.

The previous steps are repeated when there is a contact between two sensor nodes until each sensor node reaches the maximum utility of the photos or the sensor nodes share the same final set of photos. Given that sensor node *a* has the highest probability of delivering photos to the command center, it first starts to request the needed photos from sensor node *b* to increase the photo’s utility on its storage. Based on that, 15 nodes, which can contact the command center, upload the photos that are received from other sensor nodes inside the target area to the command center.

**Algorithm 6** Merging local K-CoverPicTrees on sensor *a*1: Tree(*a*) = Local K-CoverPicTree of sensor *a*2: Tree(*b*) = Local K-CoverPicTree of sensor *b*3: New Combined Tree(*a*) = []4: New Combined Tree(*a*) = Tree(*a*)5: **for**
*S’* = 1:size(S-common) **do**6:    Merge all child nodes of common Node(*S’*) of Tree(*b*) into the common Node(*S’*) of the New Combined Tree(*a*)7: **end for**8: Call Algorithm 4 to remove redundant photos in the New Combined Tree(*a*)9: Requested Photos from sensor *b* = Photos IDs that generated from subtracting Tree(*a*) from New Combined Tree(*a*)10: Local K-CoverPicTree of sensor a = New Combined Tree(*a*).

## 4. Performance Evaluation 

This section presents the performance evaluation of the proposed framework compared with the most related work; namely the PoIs scheme [11]. Other recent related works are discussed in Section 2. However, we could not compare our work with any of them for different reasons. For instance, in [20], the architecture of the used platform is composed of a server and a set of volunteers connected through digital television, which is implemented with a set top box connected to the television. Our work, on the other hand, focuses on resource-constrained environments. In [21,22], the PoIs are used in different context such that the server specifies the priority of the PoIs and accordingly updates the current PoI list to be forwarded to the mobile devices in each iteration. The work in [21,22] focuses on environments with dynamic change of the PoI list, and accordingly it keeps updating the current PoI list over time. Our work, on the other hand, focuses on fixed list of PoIs and it is compared against other works of similar environments.

### 4.1. System Model and Experiment Setup

**Figure 6:**
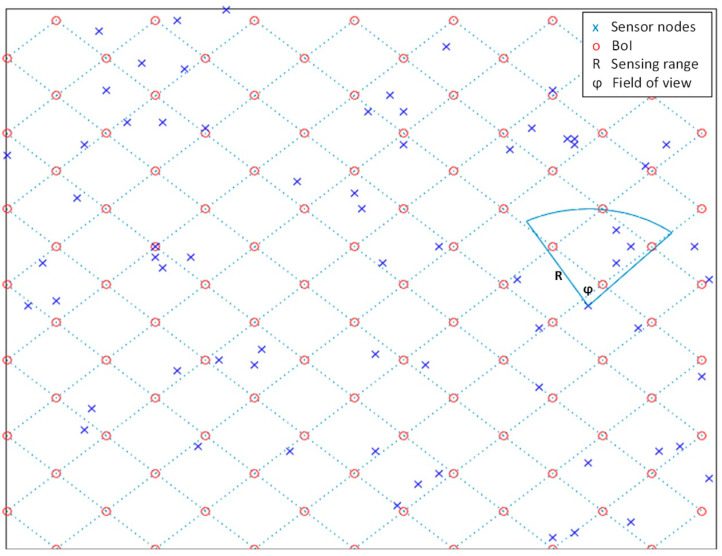
The system model of the proposed framework.

To evaluate the performance of the proposed system, it is assumed that the sensor nodes move randomly within a square target area while capturing photos of the target points of interest (i.e., BoIs) from various locations and directions. Figure 6 shows a layout of the system model. 

Any contact between two nodes results in photo sharing between them and the assumption is that, via free network links, the nodes are both able to store and exchange photos between them [11]. 

The Cambridge dataset simulates DTNs [25]. To evaluate the performance of the proposed system, the Cambridge dataset is pre-processed such that all contacts between any pair of nodes are gathered and sorted according to the contact time in ascending order. MATLAB was employed in the stimulation of DTNs. Here, 1000 nodes carry local information of photos in the network, and the location of the command center is outside the target area. In disrupting contact with the command center, the presumption is that only 15 sensor nodes, selected randomly, are able to contact the server, and thus, only 15 nodes can deliver information about the target area.

In this section, we present the selection patterns of the BoIs scheme in our proposed work and the PoIs scheme in [11]. Concerning the BoIs model, two cases are considered:

1. Case 1: The coverage disk radius, *r*, is 5 m, while the sub-regions have an area of 50 m^2^ each. Considering a target area of 100 m × 100 m, the number of sub-regions is thus 200, resulting from (100 × 100)/50. Meanwhile, the number of BoIs is 800, resulting from 200 × 4. Nonetheless, owing to redundancy between adjacent sub-region BoIs, the actual number of points is 220.
2. Case 2: The coverage disk radius, *r*, is 4 m, while the sub-regions have an area of 32 m^2^ each, resulting in 288 generated sub-regions. There are 1152 BoIs (288 × 4), but owing to redundancy between adjacent sub-region BoIs, there are 312 points.

The PoIs model does not apply boundary points to each sub-region; instead, it distributes a set of random PoIs within the target area. Hence, two cases are considered for the PoIs model, whereby in case 1, the target area is divided based on *r* = 5 m. In each sub-region, one random point is allotted and extra points are added in the target area so that case 1 would reach 220 points. For case 2, the target area is divided based on *r* = 4 m. In each sub-region, a random point is allotted, and extra points are added in the target area so that case 2 would reach 312 points. The number of photos is 500 per hour. Each time, a random direction is selected for each photo.

### 4.2. Simulation Results

In this section, the simulation results of the proposed system based on the total utility of the photos, the amount of traffic and the overall power consumption are presented. 

#### 4.2.1. Utility of the Photos

In the centralized system, which is considered as the ideal case, the photos are all transferred to a single point for processing, namely the server. This allows the selection of photos with the highest utility value. The presumption, in this case, is that all nodes are capable of contacting the server, and ideally, the nodes are in contact with the server regularly. In addition, the nodes are all assumed to have the ability to deliver the metadata of the photos to the server. Then, the metadata is processed by the server, requesting only the valuable photos. This ideal situation was used as a reference for best-case analysis when comparing the utility area of the two distributed systems (i.e., the proposed scheme and PoIs scheme) with the centralized system, which has the maximum total utility. 

**Figure 7:**
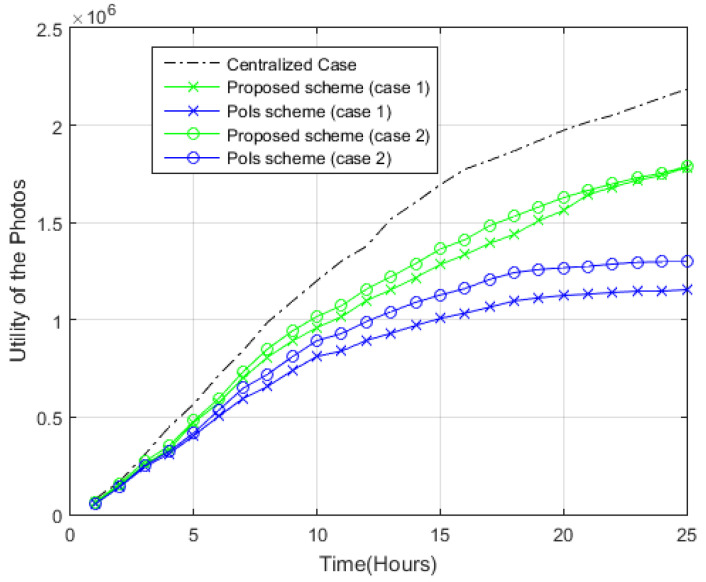
Utility of the photos.

The utility of the final selected photos of the proposed scheme and the PoIs scheme can be viewed in Figure 7. As can be observed, a longer time duration increases the number of new images generated by nodes. As there is an increasing number of sharing photos between nodes with a longer time duration, it is possible to discover photos that cover more points within the target area. The increase in time duration has thus improved the utility of the photos in both systems. As opposed to the PoIs scheme, the proposed scheme demonstrates superior performance with the total utility of higher value in both cases. For example, after 20 h in case 1, the total utility of the photos is 1.1 × 10^6^ using the PoIs scheme, while in case 2, the total utility of the photos is 1.27 × 10^6^. For both case 1 and case 2 in the proposed scheme, the utility of the photos value is approximately 1.6 × 10^6^. 

**Figure 8:**
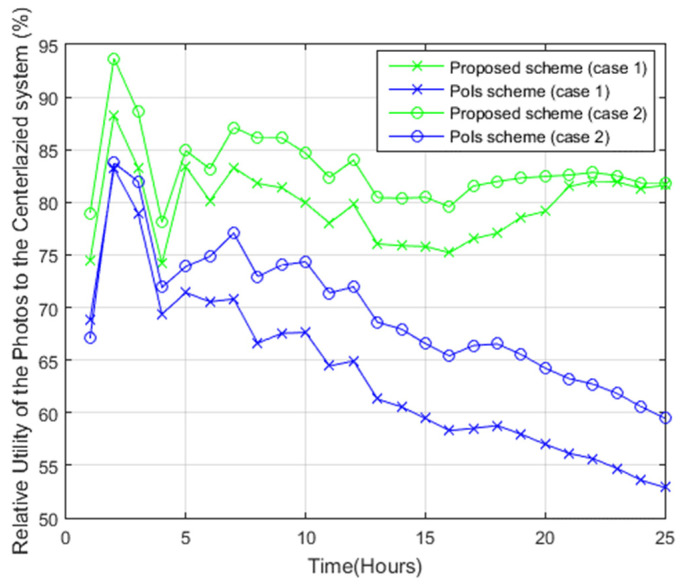
The relative utility of the photos with respect to the centralized case.

The proposed scheme and PoIs scheme were compared, in terms of efficiency, to the centralized system. As can be viewed in Figure 8, the proposed scheme attained 81% of the centralized case after 25 h, while the PoIs scheme attained 60% in case 2 and 52.5% in case 1 after 25 h.

**Figure 9:**
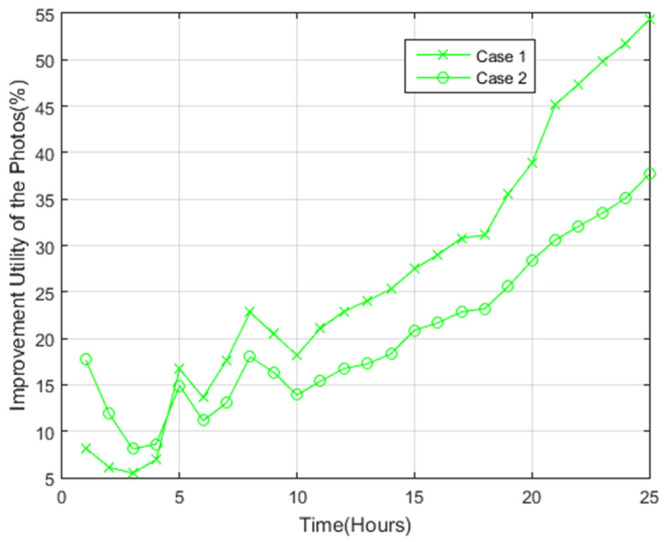
Improvement of photos utility of the proposed scheme compared to the PoIs scheme.

Figure 9 presents the improvement percentage in the photos utility of the proposed scheme compared to the PoIs scheme for case 1 and case 2. The results demonstrate that the use of the proposed scheme for case 1 led to 55% improvement after 25 h as opposed to the use of the PoIs scheme. For case 2, 37% improvement was achieved with the use of our proposed scheme as opposed to the PoIs scheme after 25 h.

#### 4.2.2. Sensor Node-to-Sensor Node Photos Exchange

**Figure 10:**
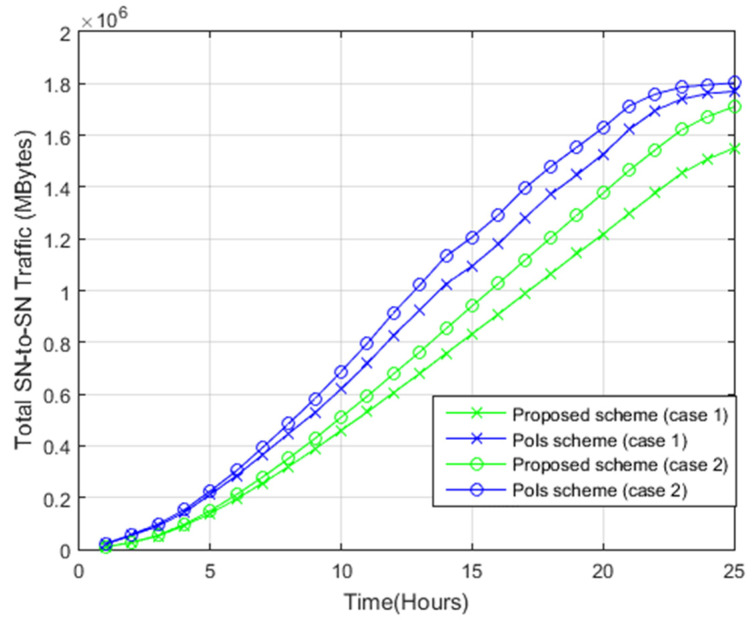
Sensor node-to-sensor node photos exchange.

Figure 10 displays the amount of sensor node-to-sensor node (SN-to-SN) transmitted photos during simulation for both systems. Unlike the PoIs scheme, the proposed scheme needs less SN-to-SN photos exchange. In the proposed system, the contact between two nodes allows the sharing of photos with common sub-region IDs only and photos of all sub-regions within the target area do not need to be exchanged between each other. In the PoIs scheme, the contact between two nodes will involve sharing of photos of all points within the target area. As such, the overall traffic of SN-to-SN photos in the PoIs scheme is higher when compared to the proposed scheme.

#### 4.2.3. Sensor Node-to-Sensor Node Metadata Exchange

**Figure 11:**
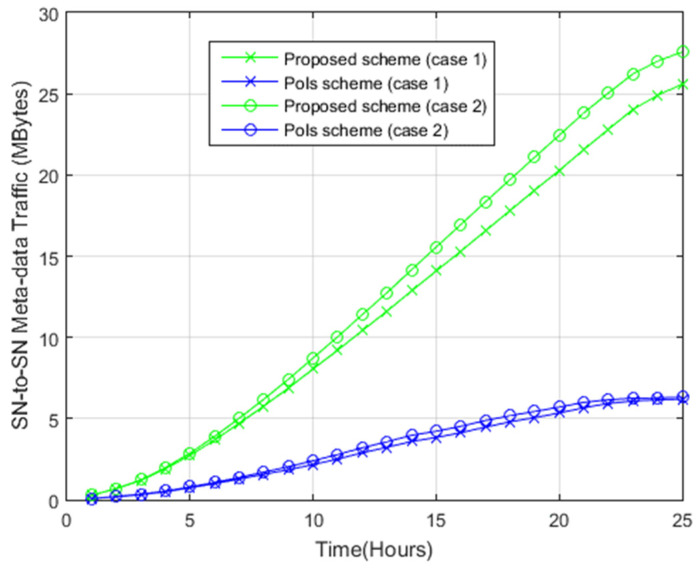
Sensor node-to-sensor node metadata exchange.

In both systems, the stored photo is merged with metadata. To avoid sharing redundant photos between nodes, the metadata of the photos were first exchanged to calculate the semantic redundancy between the photos. Figure 11 shows the metadata traffic exchanged during the simulation for both systems. For example, for case 1, the application of the proposed scheme requires 14 Mbytes at time 15, while the application of the PoIs scheme for the same case requires 4 Mbytes at time 15. For case 2, the use of the proposed scheme requires 15 Mbytes, while the use of the PoIs scheme for the same case requires 5 Mbytes at time 15. The PoIs scheme involves the metadata sharing among nodes for each photo involving four parameters of location, direction, FoV, and sensing radius. The aforementioned photo parameters are for assisting the nodes in identifying the semantic redundancy occurring between photos in the PoIs scheme. For each photo, 5 bytes are allocated for the location, 2 bytes for the direction, 2 bytes for the FoV, and 1 byte for the sensing radius. As such, the size of the control message (metadata) becomes the number of photos sent to each node multiplied by 10 bytes for each photo. The Photo ID involves FoV, direction, sensing radius, and location. Each photo ID is 10 bytes in size. In the proposed system, the contact between two nodes involves sharing four features about each photo inside the clustering tree (sub-region ID, BoIs ID, sector ID, and Photo ID). Two Bytes are allocated to the sub-region attribute, one byte for the BoIs attribute, and one byte for the sector attribute. The Photo ID involves FoV, direction, sensing radius, and location. Each photo ID is 10 bytes in size. For example, assume that photo *a* has the features {(1, 1, 1) (1, 2, 1) (1, 3, 1)}. Accordingly, the following metadata traffic will be shared {(1, 1, 1, *a*), (1, 2, 1, *a*), (1, 3, 1, *a*)}. Thus, the size of the metadata is (2 + 1 + 1 + 10) × 3. Assume the number of photos is *N*, and the number of features set of each photo is *F*. The total size of metadata traffic (in bytes) is:

(4) $$S_{\mathrm{metadata}}=\sum_{p=1}^{N}(14\times F_{p})$$

where *p* represents the photo number.

Hence, the proposed system has higher SN-to-SN metadata traffic compared with the PoIs scheme as the proposed system involves sharing more features about each photo compared to the PoIs scheme.

#### 4.2.4. Sensor Node-to-Command Center Photos Transfer

To simulate emergency situations, the presumption is that only 15 nodes are allowed to make contact with the command center. Hence, the amount of transferred photos to the command center in both systems is presented in the simulation.

**Figure 12:**
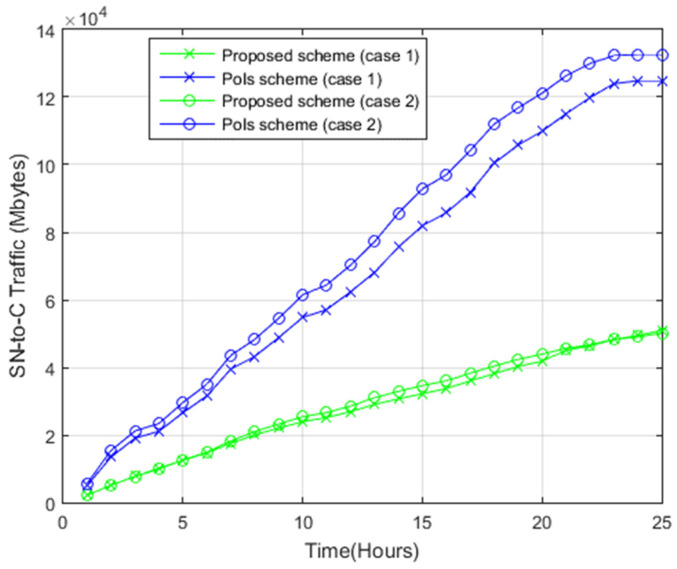
Sensor node-to-command center photos transfer.

Figure 12 shows the ratio of the overall transferred traffic from 15 nodes within the DTN to the command center in both systems. The PoIs scheme has more points of interest, translating into more transferred photos to the command center. Hence, case 2 has higher total sensor node-to-command center (SN-to-C) traffic as opposed to case 1. Comparatively, for the proposed system for case 2, higher SN-to-C traffic was reported, as opposed to case 1, because case 2 contains more sub-regions, which means more transferred photos to the server.

As opposed to the PoIs scheme, the proposed scheme has smaller SN-to-C traffic because the proposed system allows sharing only photos with common sub-regions between nodes, as each node shares with other nodes. On the other hand, in the PoIs scheme, all the nodes share photos of all PoIs within the target area with other nodes. As such, the PoIs scheme has more photos in each node as opposed to the proposed scheme.

#### 4.2.5. Total Data Transfer

**Figure 13:**
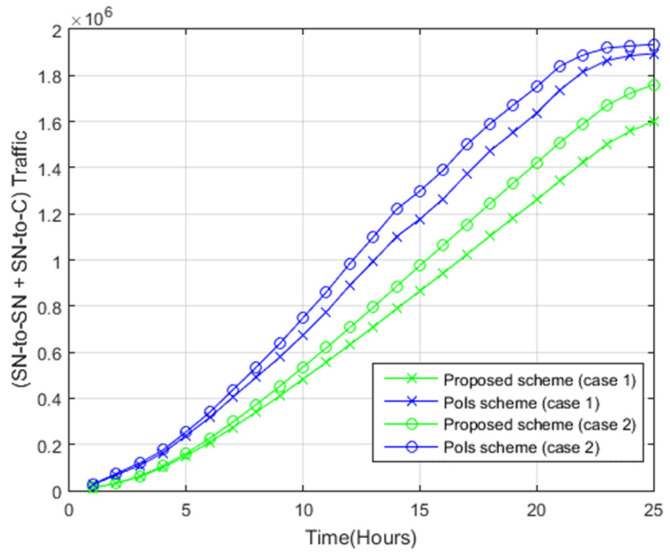
Total data transfer.

Figure 13 shows the total data transfer (SN-to-SN and SN-to-C traffic) in both systems. Unlike the PoIs scheme, the proposed scheme needs less total data transfer, owing to the fact that the proposed system reported lower SN-to-SN and SN-to-C traffic transfer. For instance, for case 1, the application of the proposed scheme requires 1.6 × 10^6^ Mbytes at time 25, while the application of the PoIs scheme for the same case requires 1.9 × 10^5^ Mbytes at time 25. For case 2, the use of the proposed scheme requires 1.8 × 10^6^ Mbytes, while the use of the PoIs scheme for the same case requires 1.9 × 10^6^ Mbytes at time 25.

**Figure 14:**
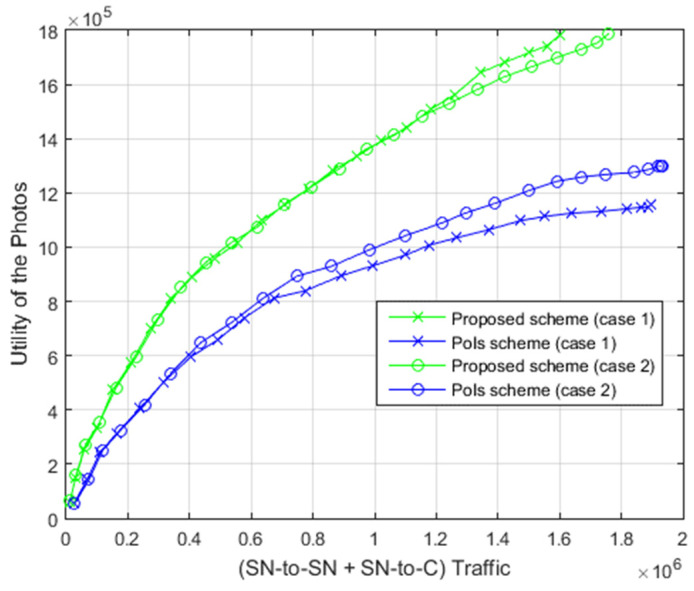
Relation between total data transfer and photos utility.

The relationship between the total data transfer and the utility of the photos is displayed in Figure 14. As shown clearly, the proposed scheme attained superior utility of the photos with a similar amount of total data transfer as opposed to the PoIs scheme.

**Figure 15:**
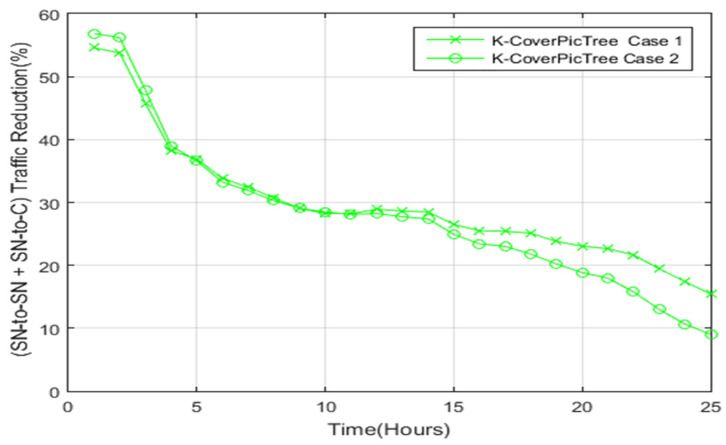
Total traffic reduction of the proposed scheme compared to the PoIs scheme.

Figure 15 highlights the reduction ratio of the SN-to-SN and SN-to-C traffic of the proposed system as opposed to the PoIs scheme. After 5 h, the proposed system had a reduction ratio of 35% as opposed to the PoIs scheme. Then, after 15 h, the reduction ratio was 25%. After 25 h, case 1 showed a reduction ratio of 15%, while case 2 had a reduction ratio of 10% compared to the Pols scheme. Case 1 has a higher reduction ratio when compared to case 2 because case 2 has a higher number of sub-regions that require the additional overhead of photos exchange.

#### 4.2.6. Power Consumption

The power consumption of the nodes within the network was evaluated through the radio power model applied in the LEACH protocol [26]. The energy consumption of the transmitter $\left(E_{Tx}\right)$ and the receiver $\left(E_{Rx}\right)$ to send a *k*-bit packet can be calculated using the following equations:

(5) $$E_{Tx}\left(k,d\right)=\left(E_{\mathrm{elec}}\times k\right)+\varepsilon_{\mathrm{amp}}\times k\times d^{2}\;,\;\;\;d<d_{0}\;$$

(6) $$E_{Tx}\left(k,d\right)=\left(E_{\mathrm{elec}}\times k\right)+\varepsilon_{\mathrm{amp}}\times k\times d^{4}\;,\;\;\;d\geq d_{0}\;$$

(7) $$E_{Rx}\left(k\right)=E_{\mathrm{elec}}\times k$$

where *E_elec_* = 50 nJ/bit, $\varepsilon_{\mathrm{amp}}$= 100 pJ/bit/m^2^, and *d*_0_ = 86.2 m.

**Figure 16:**
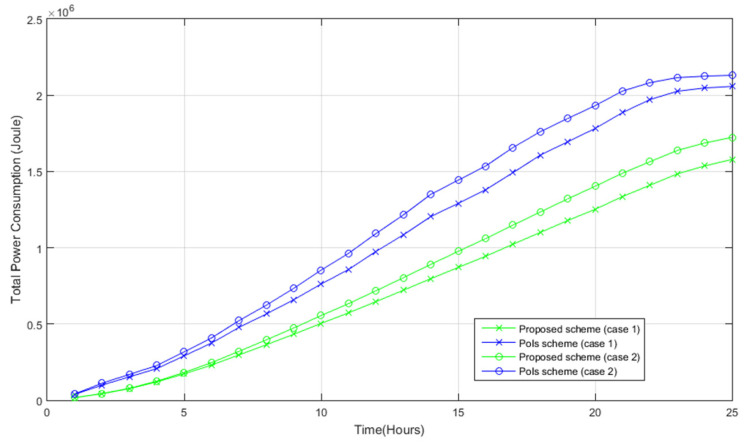
Total power consumption.

We assume the location of the command center is outside the target area (the distance between nodes and the command center is greater than 100 m). Next, the total power consumption of SN-to-SN traffic and SN-to-C traffic for both systems is discussed. Figure 16 shows the total power consumption for both systems, whereby the power consumption is akin to the total (SN-to-SN and SN-to-C) traffic in Figure 13. As evidenced, the proposed system consumes less power, owing to the fact that the proposed system reported lower SN-to-SN and SN-to-C traffic. Hence, the proposed system is superior to the PoIs scheme in terms of power consumption and the utility of the photos in general; specifically, it consumes less power and has a higher utility of the photos.

## 5. Conclusions

Designing an effective distributed visual crowdsensing methodology in disaster relief and emergency situations are considered as a challenging task. Such methodology should adequately address several constraints, including limited bandwidth, limited energy resources, and interrupted communication links with the command center or server. This is because visual data requires more data processing and more bandwidth to transmit than scalar data. To solve this issue, this paper presents a distributed visual crowdsensing photo selection system for area coverage that overcomes these issues. The performance of the proposed scheme was evaluated and compared against the state-of-the-art in terms of the utility of the photos, overall traffic transfer, and overall energy consumption. In terms of the utility of the photos, the proposed scheme can find photos with a higher utility value. The improvement ratio of the total utility is more than 38%. In terms of traffic transfer, it was found that the proposed scheme needs to exchange less amount of traffic among sensor nodes, as well as between the sensor nodes and the command center. The overall traffic reduction is more than 20%. In terms of energy consumption, the overall reduction is more than 25%. It was discovered that transferring pictures between sensor nodes, as well as between sensor nodes and the command center, requires less power usage in the system.

## Data Availability

Not applicable.
